# S-Adenosylmethionine: A Multifaceted Regulator in Cancer Pathogenesis and Therapy

**DOI:** 10.3390/cancers17030535

**Published:** 2025-02-05

**Authors:** David Fernández-Ramos, Fernando Lopitz-Otsoa, Shelly C. Lu, José M. Mato

**Affiliations:** 1 Precision Medicine and Metabolism Lab, Center for Cooperative Research in Biosciences (CIC bioGUNE), Basque Research and Technology Alliance (BRTA), 48160 Derio, Spain; dfernandez.ciberehd@cicbiogune.es (D.F.-R.); flopitz@cicbiogune.es (F.L.-O.); 2 Centro de Investigación Biomédica en Red de Enfermedades Hepáticas y Digestivas (CIBERehd), Instituto de Salud Carlos III, 28029 Madrid, Spain; 3 Karsh Division of Gastroenterology and Hepatology, Cedars-Sinai Medical Center, Los Angeles, CA 90048, USA; shelly.lu@cshs.org

**Keywords:** S-adenosylmethionine, methylation, cancer, therapy, biomarker

## Abstract

S-adenosylmethionine (SAMe) is a key methyl donor that plays a critical role in a variety of cellular processes, such as DNA, RNA and protein methylation, essential for maintaining genomic stability, regulating gene expression and maintaining cellular homeostasis. The involvement of SAMe in cancer pathogenesis is multifaceted, as through its multiple cellular functions, it can influence tumor initiation, progression and therapeutic resistance. In addition, the connection of SAMe with polyamine synthesis and oxidative stress management further underscores its importance in cancer biology. Recent studies have highlighted the potential of SAMe as a biomarker for cancer diagnosis and prognosis. Furthermore, the therapeutic implications of SAMe are promising, with evidence suggesting that SAMe supplementation or modulation could improve the efficacy of existing cancer treatments by restoring proper methylation patterns and mitigating oxidative damage and protect against damage induced by chemotherapeutic drugs. Moreover, targeting methionine cycle enzymes to both regulate SAMe availability and SAMe-independent regulatory effects, particularly in methionine-dependent cancers such as colorectal and lung cancer, presents a promising therapeutic approach. Additionally, exploring epitranscriptomic regulations, such as m6A modifications, and their interaction with non-coding RNAs could enhance our understanding of tumor progression and resistance mechanisms. Precision medicine approaches integrating patient subtyping and combination therapies with chemotherapeutics, such as decitabine or doxorubicin, together with SAMe, can enhance chemosensitivity and modulate epigenomics, showing promising results that may improve treatment outcomes. This review comprehensively examines the various roles of SAMe in cancer pathogenesis, its potential as a diagnostic and prognostic marker, and its emerging therapeutic applications. While SAMe modulation holds significant promise, challenges such as bioavailability, patient stratification and context-dependent effects must be addressed before clinical implementation. In addition, better validation of the obtained results into specific cancer animal models would also help to bridge the gap between research and clinical practice.

## Simple Summary

S-adenosylmethionine (SAMe) is a key molecule involved in cellular methylation and metabolic processes. It plays a crucial role in DNA, RNA and protein methylation, as well as in pathways like polyamine synthesis and transsulfuration. Dysregulation of SAMe metabolism is linked to various cancers, where its depletion leads to genomic instability, abnormal gene expression and tumor progression. Studies suggest SAMe could be a useful cancer biomarker and may enhance treatment by restoring methylation balance, reducing oxidative damage and protecting cells from chemotherapy effects. Targeting SAMe-related pathways, including combination with anticancer drugs, could offer new therapeutic options. Despite its potential, clinical applications remain challenging due to variability in response, poor bioavailability and the complexity of SAMe’s effects across different cancer types. Future research should focus on improving SAMe delivery, identifying biomarkers for patient-specific treatments and integrating SAMe with existing therapies. With further clinical validation, SAMe could become a valuable tool in personalized cancer treatment, helping to improve outcomes and expand therapeutic options.

## 1. Introduction

S-adenosylmethionine (SAMe) is a simple molecule, present in all living cells and synthesized in just one step by the coupling of L-methionine with the adenosyl moiety of adenosine triphosphate (ATP) [1,2]. Since its discovery by Giulio Cantoni in 1951, SAMe has been extensively studied because of its role as the main biological methyl donor, since its methyl group is easily displaced by a nucleophilic substrate to form S-adenosylhomocysteine (SAH) [3]. This review explores the role of SAMe in cancer establishment and development, as well as its potential as a biomarker and therapeutic target.Overview of S-Adenosylmethionine Synthesis

**Figure 1:**
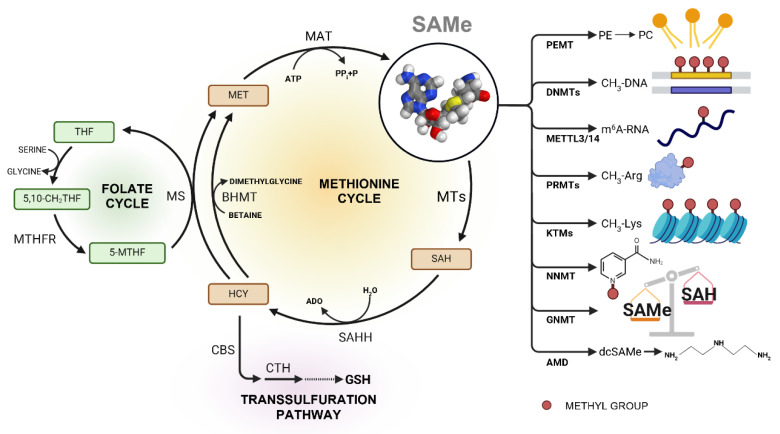
Overview of the methionine cycle, and connections with folate cycle, and transsulfuration pathway illustrating the role of SAMe in key cellular processes including nucleic acids, protein and lipid methylation, SAMe/SAH homeostasis and polyamine synthesis. SAMe molecule structure obtained from: https://3d.nih.gov/doi/11913/2 (accessed on 16 December 2024). Gray: carbon; blue: nitrogen; red: oxygen; yellow: sulfur; white: hydrogen. ADO: adenosine; AMD: adenosylmethionine decarboxylase; BHMT: betaine HCYmethyltransferase; CBS: cystathionine beta-synthase; CTH: cystathionase; DNMTs: DNA methyltransferases; GNMT: glycine N-methyltrasferase; GSH: glutathione; KTMs: lysine-specific methyltransferases; MAT: methionine adenosyltransferase; METTL3/14: methyltransferase-like protein 3/14; MS: methionine synthase; MTs: methyltransferases; MTHFR: methylenetetrahydrofolate reductase; NNMT: nicotinamide N-methyltransferase; PC: phosphatidylcholine; PE: phosphatidylethanolamine; PEMT: phosphatidylethanolamine N-methyltransferase; PRMTs: protein arginine methyl transferases; SAH: S-adenosylhomocysteine; SAHH: S-adenosylhomocysteine hydrolase; SAMe: S-adenosylmethionine.

Focusing on mammals, although being synthesized in all cells, liver is the organ where most SAMe is produced, accounting for about 85% of transmethylation reactions and 50% of methionine metabolism [4,5]. SAMe is produced via the enzyme methionine adenosyltransferase (MAT) (Figure 1), which depending on the tissue, corresponds to three distinct forms: MAT I and MAT III, codified by the gene *MAT1A*, and MAT II, encoded by the gene *MAT2A* [6]. *MAT1A* is mainly expressed in the adult and differentiated liver [7].

The α1 subunit produced by *MAT1A* organizes both into dimers (MAT III) and tetramers (MAT I) [8]. *MAT2A* gene is expressed in extrahepatic tissues, in the fetal and proliferating liver, and in liver disease, producing the α2 subunit that adopts a tetrameric disposition (MAT II) [7,8]. Although sharing an amino acid identity of 84%, MAT II has the lowest K_m_ for methionine (4–10 μM) whereas MAT III has the highest K_m_ (215 μM–7 mM) and MAT I shows an intermediate K_m_ (23 μM–1 mM). The activity of MAT enzymes is also modulated by the product of the catalytic reaction, SAMe. Normal cellular concentration of SAMe can inhibit MAT II strongly (IC50 = 60 μM), and minimally MAT I (IC50 = 400 μM) and even stimulates MAT III up to 8-fold at 500 μM SAMe [8,9,10,11]. This enzymatic diversity allows the cell to precisely regulate SAMe synthesis under variable physiological conditions, from SAMe production at low methionine levels by MAT II to the handling of very high methionine concentrations by MAT III, even avoiding possible toxicity. Likewise, inhibition by its product allows the intracellular homeostasis of SAMe.

SAMe constitutes the first product of the “methionine cycle” that interacts with the polyamine synthesis, the transsulfuration pathway and the folate cycle, in addition to participate in a wide variety of transmethylation reactions [12,13].

### 1.1. Importance of SAMe in Cellular Processes

Considering the wide variety of biochemical reactions in which it participates (Figure 1), as well as the numerous pathways with which it interacts, it is easy to understand the importance of SAMe for fundamental cellular processes.

#### 1.1.1. Methylation Reactions

As mentioned above, the main and most studied function of SAMe is to participate in methylation reactions. The substrates to which SAMe is able to transfer its methyl group are varied, including DNA, RNA, proteins, small molecules or lipids, depending on the specific methyltransferase involved in the reaction.

##### Nucleic Acids Methylation

Through DNA methylation, which involves DNA methyltransferases (DNMTs), the methyl group of SAMe is transferred to cytosine at CpG residues modulating the access of transcription factors to chromatin and the basal transcriptional machinery. Changes in DNA methylation are crucial during processes such as genomic imprinting, embryogenesis, aging, X chromosome inactivation, and cancer [14,15,16,17].

Continuing with nucleic acids modifications, RNA methylation appears as the most common modification in the interior of mRNA, mainly mediated by methyltransferase-like protein 3 and 14 (METTL3 and METTL14) that convert adenosine to N^6^-methyladenosine (m^6^A). mRNA methylation is emerging as a key mechanism involved in translation, splicing, export and degradation, and dysregulations in the process lead to the development of various diseases, including melanoma, leukemia, pancreatic and liver cancer, etc. [18,19,20,21,22,23].

##### Protein Methylation

Protein methylation, both in arginine and lysine residues, promotes changes in their functionality, affecting processes such as signal transduction, ribonucleoprotein export, transcription and splicing, among others [24]. Protein arginine methyl transferases (PRMTs) constitute a family of enzymes capable of transferring SAMe methyl group to arginines, modifying them as monomethylarginines, symmetric or asymmetric dimethylarginines. Nine PRMTs have been identified, acting on a plethora of proteins corresponding to histones, signal receptors, transcription factors, coactivators, RNA-binding proteins, etc. [25]. With regard to lysine methylation, it mainly occurs on histones, components of the cellular protein synthesis machinery, mitochondrial proteins, and molecular chaperones by the action of lysine-specific methyltransferases (KTMs) resulting in mono, di, and trimethylated Lys [26,27]. Given the wide variety of biological processes affected by protein methylation, its deregulation is associated with a wide variety of diseases, including cancer.

##### Methylation of Small Molecules

SAMe serves as methyl donor in many biochemical methylation reactions that involve transferring its methyl group to small molecules, a process that is facilitated by specific methyltransferases. These reactions can take place in diverse organs and are fundamental to processes as SAMe and nicotinamide (NAM) homeostasis, creatine synthesis, epinephrine production, catecholamine deactivation and arsenic metabolism [28,29,30,31,32,33]. Importantly, it has been demonstrated that SAMe levels must be tightly controlled, and dysregulation of its homeostasis through lack of its catabolism by glycine N-methyltrasferase (GNMT) leads to liver disease and hepatocellular carcinoma (HCC), process that may be compensated by other methyltransferase activities such as NAM-methyltransferase (NNMT) [34,35,36,37].

##### Lipid Methylation

Phosphatidylcholine (PC), the major membrane phospholipid, can be synthesized from CDP-choline and phosphatidylethanolamine (PE). Particularly in the liver, both pathways act complementarily to produce the PC needed for very low-density lipoproteins (VLDL) assembly and secretion and dysregulation of PC/PE ratios can impair this process. The PE N-methyltransferase (PEMT) pathway uses SAMe to methylate PE yielding PC and being affected by both SAMe increase and depletion which ultimately can lead to dysregulation of VLDL secretion and result in liver disease, atherosclerosis and obesity [38,39,40].

#### 1.1.2. Polyamine Synthesis

Polyamines constitute a class of low-molecular-weight molecules present in all living cells and crucial to processes involving translation, transcription, cell growth and apoptosis [41]. SAMe is decisively involved in polyamine synthesis, as the production of spermidine (SPD) and spermine (SPM) from putrescine requires the sequential addition of an aminopropyl group coming from decarboxylated SAMe (dcSAMe). In fact, the rate limiting step of polyamine synthesis consists of the decarboxylation of SAMe for producing dcSAMe, in a reaction catalyzed by the enzyme SAMe decarboxylase [13]. Since polyamines’ biological functions are related with cell growth and proliferation, gene expression, protein synthesis, membrane stability and apoptosis regulation, their relationship with a wide variety of cancers has been deeply studied [42,43,44,45,46,47].

#### 1.1.3. Transsulfuration Pathway

Through transsulfuration pathway, the sulfur atom from SAMe (coming from dietary methionine) is transferred to serine to form cystathionine and then cysteine. Cysteine constitutes the rate-limiting precursor for glutathione (GSH) synthesis, particularly active in the liver and crucial in protecting cells by neutralizing reactive oxygen species (ROS), detoxifying xenobiotics and even regulating protein functions through S-glutathionylation [48]. The link between SAMe and transsulfuration pathway is double: first, the SAH produced after SAMe-dependent methylation reactions is converted into homocysteine (Hcy) by the enzyme S-adenosylhomocysteine hydrolase (SAHH). Hcy can enter the transsulfuration pathway transferring its sulfur to the serine in a reaction catalyzed by the enzyme cystathionine beta-synthase (CBS) and, subsequently, the enzyme cystathionase (CTH) cleaves cystathionine to form cysteine and alpha-ketobutyrate [49]. Second, SAMe exerts a regulatory function over CBS, since SAMe allosterically activates this enzyme controlling the flux towards transsulfuration pathway while reducing the remethylation of Hcy to methionine by inhibiting methylenetetrahydrofolate reductase (MTHFR) enzyme, depending on cellular excess or depletion of SAMe [13,49,50,51]. Defects in transsulfuration pathway, such as CBS deficiency, promote elevated Hcy, SAH and SAMe thereby destabilizing SAMe homeostasis, with diminished SAMe/SAH ratio, reduced GSH and lower PEMT activity which can promote the setting of mild steatotic liver disease (SLD) [52,53,54,55,56]. Moreover, it has been proposed that de novo cysteine synthesis via transsulfuration pathway can be fundamental to tumor growth in vivo when extracellular apport of cysteine is limited, demonstrating that the methylation status of the cell (SAMe/SAH ratio) can assist cancer progression under these conditions [57].

#### 1.1.4. Folate Cycle

As described, after conversion of SAMe to SAH in transmethylation reactions and obtention of Hcy by SAHH, the Hcy that does not flux towards the transsulfuration pathway must be remethylated to methionine, therefore closing the methionine cycle. This remethylation can be mediated by two enzymes: betaine Hcy methyltransferase (BHMT), that requires betaine and is present only in liver and kidney [58], and methionine synthase (MS) that links methionine cycle with folate cycle. MS takes the methyl group from 5-methyltetrahydrofolate (5-MTHF) and transfers it to Hcy in a reaction that yields methionine and tetrahydrofolate (THF), which reacts with serine to produce 5,10-methylene-THF (5,10-CH_2_THF) that is again converted into 5-MTHF by the enzyme MTHFR. As commented before, the amount of Hcy that is directed to the transsulfuration pathway or is remethylated depends on the SAMe/SAH ratio since high levels of SAMe activate CBS and inhibit MTHFR whereas if the SAMe level is reduced, the remethylation gets enhanced by inactivation of CBS and activation of 5-MTHF synthesis [59,60]. Importantly, the combination of BHMT and MS activities in the liver allow the maintenance of the physiological concentration of Hcy, which is in equilibrium with SAH due to the reversibility of the SAHH enzyme. Accumulation of SAH would reduce the methylation capacity of the cell, since it is a potent inhibitor of methylation reactions [60].

## 2. S-Adenosylmethionine Connections with Different Types of Cancer

**Figure 2:**
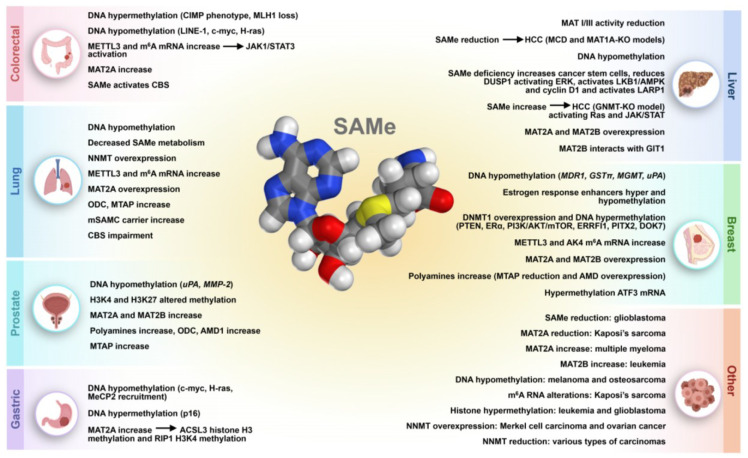
Role of S-Adenosylmethionine in various cancers. The figure highlights tissue-specific changes in SAMe metabolism, DNA methylation patterns, and the expression of key enzymes in cancers of the prostate, lung, liver, breast, colorectal, gastric, and other tissues. SAMe molecule structure obtained from: https://3d.nih.gov/doi/11913/2 (accessed on 16 December 2024). Gray: carbon; blue: nitrogen; red: oxygen; yellow: sulfur; white: hydrogen. ACSL3: acyl-CoA synthetase long chain family member 3; AK4: Adenylate kinase 4; AKT: Protein kinase B; AMD: SAMe decarboxylase proenzyme; AMD1: SAMe decarboxylase proenzyme 1; AMPK: AMP-activated protein kinase; ATF3: Activating transcription factor 3; CBS: Cystathionine beta-synthase; CIMP: CpG island methylator phenotype; DNMT1: DNA (cytosine-5)-methyltransferase 1; DOK7: Downstream of kinase 7; DUSP1: Dual-specificity MAPK phosphatase; ERα: Estrogen receptor α; ERK: Extracellular signal regulated kinase; ERRFI1: ERBB receptor feedback inhibitor 1; GIT1: G Protein Coupled Receptor Kinase Interacting ArfGAP 1; GNMT: Glycine N-methyltrasferase; GSTπ: Glutathione-S-transferase; H3K4: 4th lysine in Histone H3; H3K27: 27th lysine in Histone H3; HCC: Hepatocellular carcinoma; H-Ras: HRas proto-oncogene; JAK: Janus kinase; LARP1: La-Related Protein 1; LINE-1: Long interspersed nuclear element 1; LKB1: Serine/threonine protein kinase 11; m^6^A: N6-methyladenosine; MAT I/III: Methionine Adenosyltransferase I/III; MAT1A: Methionine Adenosyltransferase 1A; MAT2A: Methionine Adenosyltransferase 2A; MAT2B: Methionine Adenosyltransferase 2 Non-Catalytic Beta Subunit; MCD: Methionine and choline deficient; MDR1: Multidrug resistance 1; MeCP2:methyl-CpG-binding protein 2; METTL3: Methyltransferase-like protein 3; MGMT: O(6)-methylguanine DNA methyltransferase; MLH1: MutL Homolog 1; MMP-2: Matrix metalloproteinase-2; mSAMC: Mitochondrial S-adenosylmethionine carrier; MTAP: MTA phosphorylase; mTOR: Mammalian target of rapamycin; NNMT: Nicotinamide methyltransferase; ODC: Ornithine decarboxylase; PI3K: Phosphoinositide 3-kinase; PITX2: Paired like homeodomain transcription factor 2; PTEN: Phosphatase and tensin homolog; RIP1: receptor-interacting Protein 1; SAMe: S-adenosylmethionine; STAT3: Signal transducer and activator of transcription; uPA: Urokinase-type plasminogen activator.

Given the wide range of reactions in which SAMe is involved and its numerous connections to metabolic and regulatory pathways, the role of SAMe in various diseases has been explored (Figure 2). In cancer, SAMe can influence tumor initiation, progression and treatment through multiple mechanisms. Its involvement in DNA methylation, regulation of gene expression, polyamine synthesis and redox balance, among others, plays a critical role in modulating cancer cell behavior and therapeutic responses.

SAMe depletion has been identified as a common feature of many types of cancers, including liver, colon, gastric, breast, and prostate among others [9,61,62]. This trait is often associated with changes in the epigenetic status of the cancerous cells, at different levels such as DNA and histone methylation, rRNA and tRNA methylation, chromatin remodeling and methylation-dependent regulation of non-coding RNAs. SAMe depletion decreases the methylation capacity of the cell, indicated by the SAMe/SAH ratio, producing a reduction of CpG islands methylation and global DNA hypomethylation that leads to aberrant gene expression and compromises genomic stability [63]. Regarding histone methylation, SAMe depletion has been identified to favor mono-methylation of H3K9 over di-and tri-methylation which preserves heterochromatin stability [62], but also changes in H3K4 trimethylation patterns have been identified in response to modulation of SAMe levels [64]. The methylation of rRNAs, tRNAs, miRNAs and lncRNAs are also affected by SAMe depletion producing changes in ribosome biogenesis, protein synthesis and gene regulation [65,66]. Although these mechanisms appear to be common to many types of cancer, the numerous studies conducted on the relationship between SAMe and cancer show particular mechanisms by which it may exert its influence.

### 2.1. Liver Cancer

As the main organ involved in SAMe synthesis and catabolism, together with the interconnections with polyamine synthesis, the transsulfuration pathway, the folate cycle and the varied methylation reactions, all essential in the liver, it is not surprising that SAMe homeostasis must be tightly controlled regardless of the different nutritional conditions or requirements of the organism. Misregulation of hepatic SAMe metabolism, particularly if chronically maintained over time, results in metabolic dysfunction-associated steatotic liver disease (MASLD), previously known as non-alcoholic fatty liver disease (NAFLD) and defined as the presence of hepatic steatosis in conjunction with one cardiometabolic risk factor and no other discernible cause [28,34,37,67,68,69,70]. MASLD can progress from isolated hepatic steatosis (IHS) to metabolic dysfunction-associated steatohepatitis (MASH) that includes inflammation and cellular injury and, in a percentage of the cases, can progress to cirrhosis, HCC and mortality [71,72,73,74]. According to 2022 data, primary liver cancer (comprising HCC and intrahepatic cholangiocarcinoma) ranked as the sixth most frequently diagnosed cancer worldwide, and the third leading cause of cancer-related deaths after lung and colorectal cancers [75]. HCC comprises about 75–85% of primary liver cancer cases, and the trend in western countries is for MASH to overtake viral hepatitis as the leading cause of HCC [76,77,78,79]. The evidence about SAMe deficiency as a risk factor for HCC development has been recognized over years, since there is a reduction in MAT I/III activity in hepatoma cell lines, in murine HCCs, in human liver cirrhosis and HCCs and in preneoplastic and neoplastic livers [80,81].

One of the first clues implicating methionine metabolism in liver disease emerged in 1932, when Best demonstrated that a diet deficient in methyl groups (methionine, choline and folates) produced hepatic steatosis in rats [82,83]. Moreover, restriction of methionine and choline intake in mice and rats has been employed over the years as a useful animal model in the study of MASH, leading to the development of steatohepatitis, fibrosis and even HCC if sustained over time and combined with high fat diet (HFD) [84,85,86,87,88,89,90,91,92,93]. The mechanisms by which reduction of methionine and choline in the diet promote liver injury and ultimately HCC are closely linked to SAMe metabolism.

As a murine model of MASH, methionine and choline dietary restriction has been widely used over time. Traditionally, the methionine and choline deficient (MCD) diet, with total lack of both nutrients, was shown to produce steatosis, cell death, inflammation, oxidative/ER stress, and fibrosis, with severe weight loss in the absence of insulin resistance [94,95]. To avoid severe weight loss, the model was slightly modified by maintaining choline deficiency but including 0.1% methionine. This improved model, named choline-deficient L-amino acid-defined (CDAA) diet (or simply 0.1MCD), avoids weight loss but maintains similar characteristics as the MCD diet [96,97,98]. Chronic feeding with this diet results in development of adenomas and HCC related with fibrosis and oxidative DNA damage [91]. Mechanistically, feeding the 0.1MCD diet produces a reduction in SAMe hepatic concentration, together with an increase in SAH that leads to a marked decrease in SAMe/SAH ratio. This lowered methylation potential of the cell inhibits methylation reactions, including PEMT flux which impairs the synthesis of PC and, therefore, the secretion of VLDL promoting triglyceride (TG) accumulation [99]. Moreover, the first enzyme in transsulfuration pathway, CBS, is reduced after 0.1MCD diet corresponding with a diminished GSH and GSH/GSSG ratio, biomarker of oxidative stress. Hepatic TG accumulation, oxidative stress, occurrence of oxidized species of fatty acids and lipotoxicity, all of them maintained over time, are proposed to be the main promoters of HCC in this model.

For demonstrating the central importance of SAMe in MASLD, MASH and HCC progress, *Mat1a* knockout (KO) mice model was developed. Deletion of *Mat1a* gene in mice promotes chronic SAMe reduction in the liver, that leads to hepatic hyperplasia at 3 months of age, intrahepatic fat accumulation, MASH and fibrosis at 8 months and, spontaneous HCC by 18 months of age [68,69,80,100]. Similarly to MCD diet, these mice present reduced SAMe/SAH ratio, diminished PEMT flux and lower VLDL secretion, together with oxidative stress, reduced transsulfuration pathway and dyslipidemia. This model has offered crucial insights into the pathogenesis of HCC in the context of chronic SAMe deficiency. Importantly, DNA hypomethylation has been found in this animal model suggesting an epigenetic regulation, which also correlates with advanced forms of MASLD in patients [101], and may generate genomic instability throughout carcinogenic processes. This kind of alteration has been linked to numerous human HCCs during their development and, moreover, the apurinic/apyrimidinic endonuclease 1 (APEX1) that should protect cells against genomic instability, was found to be downregulated in *Mat1a* KO mice in a SAMe-dependent manner suggesting an important role of DNA hypomethylation in the development of HCC [100,102].

Another mechanism by which SAMe deficiency can promote HCC development in *Mat1a* KO mice is constituted by the increase of hepatic cancer stem cells. In the liver, oval cells constitute the stem cells, reside near the bile ducts and are normally quiescent. Under liver injury and in models of hepatocarcinogenesis, oval cells can activate and start proliferating. Interestingly, diets deficient in methyl groups have been used to induce the growth of oval cells [103,104], and *Mat1a* KO mice have a population of CD133^+^ CD49f^+^ oval cells with increased mitogen-activated protein kinase (MAPK) and extracellular signal regulated kinase (ERK) pathways that possess tumorigenic potential resembling cancer stem cells and were insensitive to transforming growth factor-β (TGF-β) apoptotic effect [100,105,106,107].

There is a third process involved in HCC onset in *Mat1a* KO mice through an ERK pathway misregulation. In particular, *Mat1a* KO mice show a reduced expression, both at mRNA and protein level, of the enzyme dual-specificity MAPK phosphatase (DUSP1) that inhibits ERK in liver cells [108]. Additionally, in human HCC DUSP1 abundance is directly correlated with apoptosis but inversely to proliferation index [109]. The mechanism whereby SAMe regulates DUSP1 in *Mat1a* KO mice is double: at the mRNA level deficiency of SAMe decreases p53 binding to *DUSP1* promoter reducing its transcription, and at the protein level low SAMe amount increases DUSP1 ubiquitination and proteasomal degradation [108].

Another signaling pathway affected by *Mat1a* reduction is the axis serine/threonine protein kinase 11 (LKB1)/AMP-activated protein kinase (AMPK)/endothelial nitric oxide synthase (eNOS). In the liver, this cascade is activated by hepatocyte growth factor (HGF) and blocked by SAMe. Activation of AMPK promotes cytoplasmic translocation of the RNA binding protein Hu antigen R (HuR) that stabilizes mRNA of cell cycle proteins such as cyclin A2 and D1 [110,111]. Consequently, mice lacking *Mat1a* and chronic SAMe deficiency present a basal activation of LKB1 and AMPK, cytoplasmic HuR, cyclin D1 expression and proliferation [110]. In addition, the study of a *Mat1a* KO-derived HCC cell line (SAMe-D) demonstrated that LKB1 regulates Akt (protein kinase B)-mediated survival through its hyperphosphorylation and also blocks the apoptotic response through phosphorylation and retention of p53 in the cytoplasm [112].

The analysis of the phospho-proteome in *Mat1a* KO mice livers revealed a remarkable hyperphosphorylation of La-Related Protein 1 (LARP1), associated also with overexpression of this protein [113]. In this context of SAMe deficiency, cyclin-dependent kinase 2 (CDK2) is able to phosphorylate LARP1 in T449. LARP1 gets activated after phosphorylation, which induces translation of 5′-terminal oligopyrimidine (TOP)-containing mRNAs. *Mat1a* KO livers present induction of several TOP proteins, including the oncogenic proteins RPS3 and RPL18. Both LARP1 and TOP proteins are induced in human MASH and HCC [113].

So far, we have seen the effect on hepatic carcinogenesis of a reduced level of SAMe, however, as mentioned above, SAMe must be finely regulated by avoiding both its depletion and accumulation. In the liver, the enzyme GNMT is responsible for eliminating excess SAMe and maintaining a constant SAMe/SAH ratio. While a low SAMe/SAH ratio is able to inhibit methylation reactions, an abnormally high ratio can lead to aberrant methylations [13,114]. GNMT reduction has been detected in human HCC and in the liver of patients with alcohol-induced cirrhosis and infected with hepatitis C virus who are at risk of developing HCC [115,116,117]. Additionally, mild liver injury with elevated transaminases and SAMe levels has been detected in individuals harboring mutations in the *GNMT* genetic sequence [118,119]. In line with these findings, *Gnmt* KO murine model shows an increase of 35-fold in the hepatic SAMe content and a 100-fold increase in SAMe/SAH ratio. These mice spontaneously develop liver steatosis and fibrosis at 3 months of age and HCC by 8 months [37], but the mechanism involved in carcinogenesis is different from the one found in the case of *Mat1a* KO mouse. The supraphysiologic level of hepatic SAMe level in the *Gnmt* KO mice resulted in hypermethylation of the promoter regions of Ras and Janus kinase (JAK)/signal transducer and activator of transcription (STAT) inhibitors (Ras-association domain family/tumor suppressor (RASSF) 1 and 4 and suppressor of cytokine signaling (SOCS) 1–3 and cytokine-inducible SH2-protein), resulting in decreased expression of these inhibitors and increased expression and activity of Ras and JAK/STAT pathways [37]. The activation of Ras/MEK/ERK and JAK/STAT pathways are essential for human HCC onset, and suppression of SOCS and RASSF has been proposed as the mechanism [13,120]. Additionally, the study of a cell line isolated from the tumor of *Gnmt* KO mice linked also the activation of the Ras pathway with an increase in LKB1/AMPK signaling [35].

*MAT1A* and *GNMT* are expressed in the adult liver and constitute markers of a healthy normal liver. Whereas a reduction in both enzymes is associated with liver injury and HCC development, an increase in the expression of *MAT2A* and *MAT2B* genes has been demonstrated. As previously described, *MAT2A* is mainly expressed in extrahepatic tissues, fetal liver and during processes that imply cell proliferation such as liver regeneration [6,121]. *MAT2B* gene codifies for a catalytic subunit that associates with MATII enzyme, is expressed in extrahepatic tissues and during liver development [122,123]. In HepG2 cells, *MAT2A* and *MAT2B* are induced by leptin which correlates with the mitogenic potential of the cells in a mechanism involving increase of polyamine synthesis and ERK, STAT and PI3K (Phosphoinositide 3-kinase) pathways activation [124,125]. *MAT2A* overexpression in liver and colon cancer increases tumor survival and chemo-resistance, by a mechanism involving B-cell lymphoma 2 (*BCL-2*) transcription, acting directly as a transcription factor that transactivates the *BCL-2* promoter, and also stabilizing the BCL-2 protein [126]. Interestingly, *MAT2B* exists in two isoforms, V1 and V2, and *MAT2B* V1 acts as a NF-κB-dependent survival factor in liver cancer cells [123]. Studies of MAT2B interactome revealed both variants can interact with HuR and modulate subcellular localization of HuR, promoting its translocation to the cytosol where it stabilizes proliferation-related targets such as cyclin D1 and A [127]. In addition, both MAT2B splicing variants are able to interact with G Protein Coupled Receptor Kinase Interacting ArfGAP 1 (GIT1) recruiting and activating mitogen-activated protein kinase kinase (MEK) and ERK promoting growth and tumor formation [128], and also activating Ras/Raf [129]. MAT2B and GIT1 are frequently overexpressed in human liver and colon cancers, constituting a mechanism of growth and metastasis in these types of cancers [128].

### 2.2. Breast Cancer

Breast cancer is one of the most prevalent malignancies among woman worldwide, accounting 2.3 million new cases in 2022 and being the fourth leading cause of cancer-related deaths. In women, it ranks first in both cancer diagnoses and mortality [75]. The causes of breast cancer development are multiple, including environmental, genetic and epigenetic factors. Environmental risk factors include air pollution, endocrine disruptors, alcohol consumption, dietary factors, smoking and exposure to ionizing radiation [130,131,132,133,134,135,136]. Among the genetic causes, germ line mutations in the tumor suppressor genes *BRCA1* and *BRCA2*, involved in maintaining genome integrity, are the most well-known genetic risks [137,138,139], and among epigenetic factors, aberrant DNA methylations in promoter regions and histone acetylations are involved in breast cancer development and therapeutic resistance [140,141,142,143].

As the universal biological methyl donor, the role of SAMe has been intensively studied in breast cancer, both in its involvement in carcinogenesis and resistance to chemotherapy as a therapeutic drug. Epigenomic study of breast tumors cohort revealed the existence of a general hypomethylated status across the genome, particularly in large, repetitive genomic regions [144]. However, localized hypermethylation spots appear concentrated in promoter-associated CpG islands, showing also subtype-specific patterns and the existence of cumulative effects of DNA replication on genomic methylation over time [144]. These findings can explain the different methylation status shown by the different studies on breast cancer epigenetics. Due to those variations in DNA methylation depending on breast tumor subtypes, genomic localization and DNA replication-related cumulative effects, many studies seem to be controversial, with strong differences among them.

DNA hypomethylation has been found in breast cancer cell lines such as MCF-7/DOX, the doxorubicin resistant variant of MCF-7. The hypomethylated status of the promoter regions of multidrug resistance 1 (*MDR1*), glutathione-S-transferase (*GSTπ*), O(6)-methylguanine DNA methyltransferase (*MGMT*), and urokinase-type plasminogen activator (*uPA*) genes has been proposed as the mechanism contributing to chemoresistance [145]. Importantly, treatment with SAMe is able to revert the hypomethylation status of this cell line and renders this resistant cell line to radiation-induced apoptosis [146]. Similar results were found for the *uPA* gene in the highly invasive MDA-231 human breast cancer cells, with low DNA methylation reversed by SAMe supplementation [147]. Contrary to these reports, an increase in SAMe levels, together with DNMT1 up-regulation in the tamoxifen-resistant breast cancer cell line TAMR-MCF-7, was reported to lead to *PTEN* (Phosphatase and tensin homolog) promoter methylation downregulating PTEN expression and increasing Akt phosphorylation. The inhibition of DNMT1 reduced tumoral growth capacity in this cell line [148]. In fact, DNA methylation has been described in many studies involving tamoxifen resistance, identifying two main mechanisms of resistance: downregulation of estrogen receptor α (ERα) expression and abnormal activation of the PI3K/AKT/mTOR (mammalian target of rapamycin) signaling pathway [149].

Considering the estrogen receptor, hypermethylation of the estrogen receptor 1 (*ESR1*) promoter by mechanisms involving zinc finger E-box-binding homeobox 1 (ZEB1) and interleukin-1β (IL-1β) reduces its transcription and, therefore, ERα protein expression which confers resistance to tamoxifen treatments [149,150,151]. In the same line, loss of ten-eleven translocation methylcytosine dioxygenase (TET) 2 demethylase promotes tamoxifen resistance by ERα downregulation [152]. TET1 and TET3 proteins also promote the demethylation of the ubiquitin C-terminal hydrolase L1 (UCHL1) promoter, leading to increased UCHL1 expression that, in turn, downregulates ERα expression [153]. Another known mechanism of tamoxifen resistance and sensitivity involves hyper and hypomethylation of estrogen response enhancers and methylation of histones enhancing transcription of ERα target genes [154,155]. Finally, methylation status of genes upstream or downstream ERα are also related to tamoxifen resistance or sensitivity, including *p21*, Wilms’ tumor 1 (*WT1*), inhibitor of differentiation 4 (*ID4*), N-acetyltransferase type 1 (*NAT1*), elongation of very long chain fatty acids-like (*ELOVL2*), and progesterone receptor α (*PRA*) [149].

Regarding the relationship between methylation and activation of the PI3K/AKT/mTOR pathway, in addition to the mentioned effect through DNMT1 and PTEN, the methylation of genes involved in that signaling pathway inhibition has been described [149]. The hypermethylation of ERBB receptor feedback inhibitor 1 (*ERRFI1*), paired-like homeodomain transcription factor 2 (*PITX2*) and downstream of kinase 7 (*DOK7*) promoters avoid PI3K/AKT/mTOR pathway inhibition conferring chemotherapy resistance to breast cancer. Hypomethylation of the 5′UTR of activating transcription factor-3 (*ATF3*) mRNA also constitutes a mechanism of radioresistance, stabilizing its mRNA and allowing ATF3 to upregulate the phosphorylation of AKT [156]. Additionally, while the trophoblast surface antigen 2 (*TROP2*) gene typically supports cell proliferation through the PI3K/AKT/mTOR pathway, its promoter becomes methylated and silenced in tamoxifen-resistant cells, potentially involving other pathways beyond PI3K/AKT/mTOR. Also, hypermethylation of genes upstream of the pathway are involved in acquired resistance of breast cancer [149].

Other methylation-related mechanisms involved in breast cancer include m^6^A-mediated epitranscriptomic regulation of the 5′UTR sequence of adenylate kinase 4 (*AK4*) mRNA by METTL3 through ROS increase and p38 activation conferring resistance to tamoxifen [157]. Also, the long noncoding RNA H19, upregulated in TAMR-MCF-7 cell line, inhibits SAHH, which reduces DNMT3B binding and methylation of *Beclin1* activating autophagy and facilitating tamoxifen resistance [158].

As occurs in liver cancers, *MAT2A* expression appears increased in tamoxifen-resistant human breast cancers and in the TAMR-MCF-7 cell line, both compared with the tamoxifen-responsive versions. The mechanism by which *MAT2A* is overexpressed in these cells is due to the activation of NF-κB after downregulation of miR-146b, in response to PTEN reduction and Akt activation. Overexpression of MAT2A, in fact, would promote SAMe increase and *PTEN* promoter methylation, establishing a positive regulatory loop [159]. Also, *MAT2B* expression level positively correlates with cell growth and migration capacity of breast cancer cell lines and with the poor prognosis of human breast cancers [160].

Polyamines pathway is also related with breast cancer, being elevated and correlating with poor prognosis and cancer cell growth [161,162,163,164]. In polyamine synthesis, dcSAMe donates its aminopropyl group to putrescine and spermidine producing methylthioadenosine (MTA) that is rapidly cleaved to adenine and 5-methylthioribose-1-phosphate by the enzyme MTA phosphorylase (MTAP) initiating the salvage pathway that regenerates methionine [165]. MTAP is downregulated in various human cancers and appears to be deleted in many breast cancers [166]. Silencing of MTAP in a breast cancer cell line activates ornithine decarboxylase (ODC) activity elevating polyamines synthesis and promoting orthotopic xenograft tumor growth and metastasis [167].

Recently, it has been demonstrated that, in the highly aggressive basal-like breast cancer (BLBC), the SAMe decarboxylase proenzyme (AMD1) is elevated. AMD1 is the enzyme responsible for the decarboxylation of SAMe to enter in the polyamine biosynthetic pathway and in BLBC mechanisms as promoter hypomethylation, increase of the copy number of the gene and SOX10 transcriptional activity leads to its overexpression and spermidine production increase. This increased spermidine level enhances hypusination of eukaryotic initiation factor 5 A isoform 1 (eIF5A) translation factor activating transcription factor 4 (TCF4) and increasing breast cancer aggressiveness [168].

### 2.3. Lung Cancer

Lung cancer is the leading cause of cancer-related mortality worldwide, accounting for near 20% of cancer deaths. It is the first cancer for mortality and incidence for men, and the second one in woman, with smoking as the main cause and the exposure to air pollution acquiring increasing importance [75,169]. In addition to the genetic mutational changes that carcinogens promote in the lung, also changes in the inflammatory microenvironment, hypoxic stroma and epigenetic changes have been proposed as mechanisms for lung cancer onset and progression [169,170,171]. Taking this into account, SAMe involvement in lung cancer has been studied, mainly through its involvement in the epigenetic changes produced by DNMTs-mediated DNA methylation.

Whereas LKB1 behaves as a tumor oncoprotein in the liver, as mentioned earlier, it functions as a tumor suppressor in other types of cancer. In the case of lung adenocarcinomas, LKB1 loss of function has been identified associated with alterations in oncogenic pathways and mitochondrial metabolism [172]. Studies in these types of cancers have demonstrated that a large number of them present global CpG hypomethylation driven by LKB1 loss. In addition, these adenocarcinomas present decreased SAMe metabolism, repetitive element demethylation and resistance to azacytidine, a DNMT inhibitor employed as chemotherapy [173]. Authors hypothesize that depletion of SAMe is due to NNMT overexpression and overutilization of the methyl donor, which has been also observed in many cancers, including lung [174,175].

Abnormal methionine cycle has been observed in lung cancer, showing a metabolic dependence of methionine in cancer stem cells, correlating with high MAT2A expression and transmethylation rates [176]. Inhibiting MAT2A has been used to sensitize chemotherapy-resistant cells, reducing migration and proliferation and increasing apoptosis of non-small cell lung cancer due to changes in histone methylation [177]. The combination of METTL3, which is increased in non-small cell lung cancer, and MAT2A inhibitions is able to decrease m^6^A mRNA modification, leading to apoptosis [178,179].

Also, the SAMe-related pathways transsulfuration and polyamine have been linked to lung cancer. CBS, the first enzyme in the transsulfuration pathway, has been found highly elevated in lung adenocarcinoma in association with poor prognosis, while in lung squamous cell carcinomas the association is inverse [180]. Regarding the regulation of polyamine synthesis, ODC degradation has been shown to promote apoptosis in non-small cell lung cancer through a mechanism dependent on estrogen receptor [181]. Interestingly, inhibition of MTAP enzymatic activity in immunodeficient mice produces a systemic increase of the MTA concentration, which abrogates A549 and H358 xenograft tumor growth, cell lines derived from human non-small cell lung carcinoma and human bronchioalveolar non-small cell lung carcinoma cells lines, respectively [13,182].

*SLC25A26* is the human gene encoding the mitochondrial S-adenosylmethionine carrier (mSAMC). In some cancers, including non-small cell lung cancer, its expression is inversely correlated with the survival rate of the patients. The mechanism by which SAMe transport into mitochondria can affect cancers remains unclear, with the hypothesis that it may affect SAMe cytosolic abundance and regulate methylation reactions [183,184].

### 2.4. Colorectal Cancer

Colorectal cancer (CRC) is a type of cancer that starts in the colon or rectum. Overall, colorectal cancer ranks in third place in terms of incidence, and second in terms of mortality, representing about 10% of the cancer cases and deaths worldwide [75]. Among the risk factors, we can find age, family history, certain genetic mutations, diet, and lifestyle factors [185]. Aberrant DNA methylations and *MAT2A* overexpression account for the processes affected in the disease.

DNA hypermethylation leads to gene silencing in CRC patients including epigenetic inactivation of DNA mismatch repair genes. In about 15% of CRCs, it appears a phenomenon called CpG island methylator phenotype (CIMP) characterized by substantial hypermethylation of promoter CpG island sites, resulting in the silencing of several tumor suppressor genes or other tumor-related genes [186,187]. One of the genes identified to be affected by the CIMP phenotype is MutL Homolog 1 (*MLH1*), part of the mismatch repair system, whose loss promotes inability to repair strand slippage within nucleotide repeats changes the size of microsatellites, increasing risk of developing CRC [188,189,190].

Conversely, global DNA hypomethylation has been detected as an early event in CRC development [191,192,193,194]. In this case, mobile genetic elements such as long interspersed nuclear element 1 (*LINE-1*) undergo activation, being a feature of early-onset CRC and acting as factors for increased mortality [193,194,195]. This DNA hypomethylation is linked to decreased folate and SAMe levels, which correlate with reduced SAMe/SAH ratio and, therefore, lowered methylation potential in the CRCs [193,196,197]. Low methylation of promoters in CRC has also been demonstrated for the genes *c-myc* and *H-ras*, known oncogenes, which can be reversed by SAMe treatment [198].

MAT II expression and activity along the stages of CRC has also been examined, showing that MAT II was highly expressed and active in human CRCs compared to normal tissues [199]. In the same way, *MAT2A* expression in human colon cancer resections, mice polyps and cell lines were increased, correlating with epidermal growth factor (EGF), insulin-like growth factor (IGF)-I and leptin-induced proliferation. Treatment with SAMe and the polyamine pathway byproduct MTA reduced *MAT2A* expression and blocked the mitogen-activated effects [200], even inducing apoptosis by down-regulating cellular FLICE inhibitory protein (cFLIP) [201]. Moreover, SAMe and MTA treatment is also able to inhibit CRC migration, invasion and metastasis to the liver by increasing microRNA-34a/-34c/-449a expression, in a mechanism involving Notch signaling pathway inhibition [202,203].

SAMe, through its allosteric activation of the CRC abundant CBS enzyme [180,204], has been shown to increase CRC proliferation and bioenergetics. This effect is maintained overtime following SAMe treatment at 0.1 mM in HCT116 by stimulating CBS-dependent H_2_S production, which acts as signaling molecule stimulating cell line proliferation and oxygen consumption rate. On the contrary, concentrations higher than 0.1 mM leads to inhibition of HCT116 proliferation after 12 h of treatment, by a CBS-independent mechanism [205]. CBS can also directly bind to cytoskeleton modulating its organization and regulating CRC cells proliferation and migration [206].

m^6^A mRNA methylation exerted by METTL3 plays also a role in CRC progression. METTL3 overexpression in colorectal cancers compared to normal tissue has been shown to promote methylation of JAK1 transcript and STAT3 signaling pathway activation. This mechanism increased proliferation and metastasis of cancer cells in vitro and in vivo [23].

### 2.5. Gastric Cancer

Stomach cancer, including cardia and noncardia gastric cancers, ranks fifth in terms of incidence and mortality globally. *Helicobacter pylori* infection is considered the principal cause of noncardia gastric cancer, with alcohol consumption and tobacco smoking as risk factors. For the cardia gastric cancers, obesity and gastroesophageal reflux disease are the main risk factors [75]. DNA hypomethylation of the *c-myc* and *H-ras* oncogenes, together with promoter hypermethylation of the tumor suppressor p16 has been found in gastric cancer cell lines. SAMe treatment was able to revert the hypomethylated status of *c-myc* and *H-ras* promoters slowing down tumoral cells proliferation [198]. Also, hypermethylation of DNA regions in gastric cancers is able to recruit the methyl-CpG-binding protein 2 (MeCP2), which is involved in cell proliferation and apoptosis [207,208]. The overexpression of miR-22 tumor suppressor decreases MTHFR producing a reduction in SAMe level and DNA hypermethylation, reducing MeCP2 binding and, therefore, cancer cell proliferation [209].

The activation of MAT2A expression in gastric cancer protects tumoral cells from ferroptosis, by increasing methylation of histone H3 of acyl-CoA synthetase long chain family member 3 (*ACSL3*) promoter in a SAMe-dependent manner [210]. Another mechanism for MAT2A-mediated gastric cancer progression involves the regulation of anti-inflammatory functions of tumor-associated monocytes. The increase in methionine cycle due to elevated MAT2A expression upregulates receptor-interacting Protein 1 (RIP1) by H3K4 methylation at the promoter region [211].

### 2.6. Prostate Cancer

The occurrence of prostate cancer has been related to risk factors such as advanced age and certain genetic mutations. However, environmental and lifestyle-related risk factors are not as well described in a disease that ranks second in incidence and fifth in mortality among men [75]. Mechanisms involving SAMe have been described, such as DNA and histone methylation, MAT enzymes regulation and polyamines synthesis.

Examining the global gene expression of a prostate cancer cohort it was found that, in a subset of highly aggressive tumors, expression of genes controlling SAMe synthesis associate with the expression of DNA methylation targets [14]. Hypomethylation of the promoter regions of tumor-promoting genes such as *uPA* and matrix metalloproteinase-2 (*MMP-2*) have been associated with tumor growth in prostate cancer PC-3 cell line, which can be reverted by SAMe treatment [212]. Additionally to DNA CpG methylation, in the PC-3 cell line also altered histone methylation of H3K4 and H3K27 has been described after SAMe treatment, explaining the observed changes in the expression of genes [213].

Regarding MAT enzymes regulation, miR-34a, which is under-expressed in CD44^+^ prostate cancer tumors [214], and also miR-34b are able to downregulate MAT2A and MAT2B protein expression by targeting their 3′UTR. Interestingly, SAMe and MTA promote miR-34a/b expression reducing MAT2A and MAT2B abundance and inhibiting cancer metastasis [202]. Similar results have been also observed for these miRNAs in pancreatic cancer [202,215].

As in many types of cancer, polyamines also play an important role in prostate cancer. In fact, it has been demonstrated that ODC overexpression can be enough to promote prostate tumorigenesis, by altering mTOR/MAPK pathway among others [216]. mTOR pathway, in addition, is able to mediate AMD1 enzyme stability in prostate cancer, thus regulating dcSAMe production and polyamines synthesis, and elevating dcSAMe/SAMe ratio in mouse and human tumoral tissue. mTORC1 inhibition produced AMD1 reduction and abrogation of tumoral cell proliferation [217]. To avoid the decrease in the SAMe pool in tumor cells that would result from activation of the polyamine pathway, the rate limiting enzyme of the salvage pathway, MTAP, appears also upregulated and is necessary for cancer growth [218].

### 2.7. Other Types of Cancers

SAMe implication has been demonstrated in other types of cancers, mainly through DNA and histone methylation reactions.

The H3K79 histone methylation at promoters is implicated in leukemic transformation and progression. SAMe metabolism disruption reduced cell growth and induced apoptosis [219]. Similarly, in glioblastoma, mTORC1 and mTORC2 cooperate to increase SAMe production and histone H3K27 hypermethylation, promoting tumor cell survival in cellular and animal models [220]. Conversely, glioblastoma cells treated with therapeutic doses of SAMe undergo mitotic catastrophe and death [221].

Alterations of methionine metabolism and DNA methylation capacity have been also identified in highly metastatic melanoma cell lines and osteosarcoma [222,223,224], and SAMe treatment was shown to block tumorigenesis in these kind of cancers [225,226,227].

Alterations in m^6^A RNA methylation status have been demonstrated in Kaposi’s sarcoma-associated herpesvirus [228]. m^6^A modification over *MAT2A* mRNA increases its expression and, therefore, SAMe abundance rendering a reduction in lytic replication, a mechanism involved in the initiation and progression of Kaposi’s sarcoma tumors [229].

Separate mention should be made of NNMT identified alterations in expression across various types of cancers. Notably, NNMT expression is upregulated in several malignancies, including kidney renal clear cell carcinoma, kidney renal papillary cell carcinoma, pancreatic adenocarcinoma, glioblastoma multiforme, sarcoma, and lymphoid neoplasm diffuse large B-cell lymphoma [29]. High levels of NNMT expression have also been reported at both mRNA and protein levels in Merkel cell carcinoma [230] and ovarian cancer [231] as a poor prognostic feature and an advantage for cancer progression. This suggests that NNMT upregulation may be a critical factor contributing to tumor progression in these cancers, potentially enhancing tumorigenic processes such as cell proliferation, migration, and chemoresistance. In contrast, in the case of oral squamous cell carcinoma there is an inverse correlation between NNMT expression level and metastasis [232]. In other cancer types, including adrenocortical carcinoma, cholangiocarcinoma, kidney chromophobe, pheochromocytoma and paraganglioma, thyroid carcinoma and skin cutaneous melanoma, NNMT expression appears to be significantly lower [29]. These findings underscore the complex and context-dependent role of NNMT in cancer biology, where its function appears to vary widely depending on the cancer type. Several NNMT inhibitors have been identified, including methylated quinolines, nicotinamide analogues, covalent inhibitors, and amino-adenosine derived bisubstrate inhibitors [233,234,235,236]. These inhibitors could serve as valuable tools for further understanding NNMT’s role in various pathologies. Moreover, the development of potent and selective NNMT inhibitors could pave the way for novel therapeutic strategies targeting cancers associated with aberrant NNMT activity.

Finally, elevated SAMe levels have also been found to increase cell growth and viability in multiple myeloma and leukemic cells. In the case of multiple myeloma, these abnormally elevated SAMe levels have been linked to high *MAT2A* expression, and inhibiting it leads to SAMe levels decrease and impaired cell viability and proliferation [237]. Similarly, in leukemic cells, the regulatory subunit of MAT II, MAT2B, has been proposed to be responsible for SAMe level increase and higher proliferation [238].

## 3. S-Adenosylmethionine as Biomarker and Therapy

Considering the central role of SAMe in methylation processes and cellular metabolism, changes in its levels and in methionine cycle-related enzymes have been proposed as diagnostic and prognostic biomarkers. In liver cancers, including HCC and cholangiocarcinoma, MAT1A expression and activity has been found to be reduced [61] correlating with depletion of SAMe levels, promoter methylation status and, also, associates with *MAT2A* induction [121,239]. Moreover, the expression of the regulatory subunit MAT2B is frequently linked to the increase in MAT2A, conferring growth advantage to liver cancer cells [61,121,123,240] and potentially serving as biomarkers. Furthermore, SAMe is able to modulate the levels of certain lncRNAs altered in HCC, which have been proposed as biomarkers of liver cancer progression [241]. In lung cancer, plasma level of SAMe has been proposed as early detection biomarker since it is elevated in serum of patients with cancer compared to healthy individuals [242]. Finally, promoter DNA methylation status of some particular genes is used in diagnosis and prognosis of CRC [243].

In line with the broad range of cancer types in which SAMe homeostasis is impaired, its use as in anticancer therapy has been frequently explored. This includes chemoprevention, treatment and chemosensibilization in a wide type of malignancies, including hepatic, gastric, breast and colorectal cancers, among others. Table 1 summarizes SAMe roles as biomarker and therapy.

In the case of liver cancer, the reduction in SAMe content and SAMe/SAH ratio has been frequently observed during HCC onset and development, and in hepatic stages in risk of developing HCC such as cirrhotic liver. Therefore, the effect of SAMe supplementation has been widely explored in different cellular and animal models of HCC development [13,244,245,246,247,248], including preventing or reducing the occurrence and establishment of HCC foci, showing proapoptotic effects in the tumoral cells and angiogenesis decrease. However, in the case of a previously existing HCC, SAMe treatment was not able to reduce tumor size even after 24 days of intravenous infusion [117]. The proposed mechanism by which HCC was not affected by SAMe in this model is due to compensatory induction of methyltransferases (mainly GNMT) in the normal hepatic tissue to prevent supraphysiologic accumulation of SAMe. When translating the SAMe chemopreventive effect observed in animal models into clinical trial, the results were not promising. Oral SAMe administration during 24 weeks to patients with hepatitis C cirrhosis failed to reduce the HCC marker alpha-fetoprotein (AFP) serum levels in comparison with placebo group [249]. However, there were limitations with this study, including the high dropout rate and short follow-up duration. Thus, although SAMe shows potential as an anticancer therapy for liver tumors, key aspects must be addressed before its application in clinical practice. Alternatively, strategies forcing the expression of MAT1A in liver cancer, instead of directly treating with SAMe, were shown to reduce HCC growth and angiogenesis, and increase apoptosis in in vitro and in vivo models [250].

Regarding SAMe use as chemotherapy in other types of cancer, its use has been highly studied in gastric, colorectal, breast, head and neck, osteosarcoma, lung, prostate and retinoblastoma cancers, both as single treatment and in combination with other drugs as a chemosensitizing agent. The mechanisms of action by which SAMe exerts the antitumoral effects include a plethora of processes due to its varied biological activities. In breast cancer cell lines, SAMe treatment modulates the expression of miRNA-34a, miRNA-34c and miRNA-486-5p leading to caspase-dependent apoptosis and autophagy [251], but is also able to epigenetically regulate *uPA* and *MMP-2* genes by hypermethylation of CpG promoter sequences inhibiting cellular invasion and growth [147,252,253]. Interestingly, the treatment of breast cancer with SAMe in combination with the demethylating agent decitabine allows to target both DNA hypermethylation and hypomethylation of cancer-related pathways reducing tumor volume and lung metastasis [254]. The combination of SAMe with the widely employed drug doxorubicin in CG5, MCF-7 and MDA-MB 231 breast cancer cells enhances apoptotic cell death through Fas/FasL-dependent caspase 8 and 3 activation [255]. In the case of doxorubicin resistant cancer, SAMe treatment can sensitize tumoral cell lines to radiation-induced apoptosis by modifying global DNA hypomethylation profile [146]. Moreover, SAMe pro-apoptotic effects are potentiated by the autophagy inhibitor chloroquine [256], as the autophagy is frequently employed by cancer cells to escape chemotherapy.

The combination of cisplatin and SAMe exerts a synergistic effect on head and neck squamous cancer blocking cell proliferation and migration and increasing apoptosis through a mechanism involving ER-stress [257,258]. SAMe metabolism targeting has also been found to sensitize cisplatin-resistant cells in lung cancer to cisplatin treatment. In this case, both the inhibition of MAT2A and methionine restriction reduce histone methylation modulating apoptosis, DNA repair and tumor necrosis factor (TNF) pathway promoting cisplatin sensibilization [177]. Following this line, the joined inhibition of MTAP and MAT2A promotes lethality in tumor models of CRC by reducing SAMe availability and increasing MTA level, thus rising MTA/SAMe ratio and inhibiting PRMT5 activity [259]. Also, in p53 deleted colon cancer cells, SAMe treatment bypasses uL3 ribosomal protein-mediated drug resistance [260].

**Table 1:** Various mechanisms of SAMe as biomarker and therapy in different types of cancers.

Type of Cancer	Type of Role	Effect/Mechanism	References
Liver cancer (HCC, cholangiocarcinoma)	Biomarker	Reduction in *MAT1A* expression and activity	[61]
		Depletion of SAMe, reduction of promoter methylation, *MAT2A* and *MAT2B* increase	[61,121,123,239,240]
		Biomarker lncRNAs are modulated by SAMe	[241]
	Prevention	SAMe supplementation reduces HCC foci occurrence and establishment (animal model)	[117]
	Treatment	*MAT1A* forced expression reduces HCC growth and angiogenesis, and increases apoptosis in vitro and in vivo	[250]
Breast cancer	Treatment	SAMe modulates miRNA-34a, miRNA-34c and miRNA-486-5p leading to apoptosis and autophagy	[251]
		SAMe hypermethylates *uPA* and *MMP-2* genes inhibiting cellular invasion and growth	[147,252,253]
	Combined therapy	SAMe + Decitabine: targets DNA hypermethylation and hypomethylation reducing reduced tumor volume and metastasis to the lung	[254]
		SAMe + doxorubicin: enhances apoptotic cell death through Fas/FasL-dependent caspase 8 and 3 activation	[255]
		SAMe + chloroquine: inhibition of autophagy potentiates SAMe-induced apoptosis	[256]
	Sensitization	SAMe sensitizes cancer to radiation-induced apoptosis	[146]
Colorectal carcinoma	Biomarker	Promoter DNA methylation status serves as diagnosis and prognosis	[243]
	Treatment	MTAP and *MAT2A* inhibition promotes lethality in CRC blocking PRMT5	[259]
	Sensitization	SAMe treatment bypasses uL3-mediated drug resistance	[260]
Lung cancer	Biomarker	Elevated SAMe plasma level as early detection biomarker	[242]
	Sensitization	*MAT2A* inhibition sensitizes to cisplatin treatment	[177]
Head and neck squamous cancer	Combined therapy	SAMe + cisplatin: promotes ER-stress leading to apoptosis and reduced proliferation and migration	[257,258]
Gastric cancer	Treatment	SAMe hypermethylates *uPA*, c-myc and H-ras inhibiting growth	[198,261]
Glioblastoma	Treatment	SAMe induces cell cycle arrest and apoptosis and mitotic catastrophe-induced death	[221]
Osteosarcoma and prostate	Treatment	SAMe downregulates ERK1/2 and STAT3 inducing apoptosis and blocking invasion	[226,227,262]
Retinoblastoma	Treatment	SAMe inhibits Wnt2/β-catenin pathway reducing tumor growth	[263]
Various	Chemoprotection	SAMe protects against chemotherapy-induced liver injury and reduces cancer-related fatigue	[264,265,266]

In gastric cancer, SAMe is able to reverse the hypomethylated status of genes such as *uPA*, *c-myc* and *H-ras* inhibiting tumor growth, both in vitro and in vivo [198,261]. Recently, it has been also demonstrated that SAMe treatment of glioblastoma cells induces cell cycle arrest and apoptosis, together with downregulation of DNA repair mechanisms promoting mitotic catastrophe-induced death [221]. The very aggressive and highly metastatic tumor osteosarcoma shows induction of apoptosis and blocking of invasion after SAMe treatment, by downregulation of ERK1/2 and STAT3 pathways [226,227] as also happens in prostate cancer cells [262]. The Wnt2/β-catenin pathway, activated in many types of cancers and playing a significant role in its progression, is also inhibited in response to SAMe treatment in retinoblastoma showing tumor growth reduction in a xenograft animal model [263].

Finally, SAMe supplementation has been proven beneficial for reducing chemotherapy-induced side effects. In the case of drug-induced liver injury (DILI), the levels of the liver damage markers alanine transaminase (ALT), aspartate transaminase (AST) and lactate dehydrogenase (LDH) were reduced after SAMe supplementation in patients under chemotherapy treatment [264], and in resected CRC patients treated with adjuvant FOLFOX regimen, in which also reduced cancer-related fatigue [265,266].

SAMe has shown promising results in improving the efficacy of numerous chemotherapies and in various types of cancer. Advances in the study of this type of therapy, particularly in combination with other chemotherapeutics, could lead to improvements in the management of the treatment of diverse malignancies.

## 4. Conclusions and Future Perspectives

SAMe, as the main biological methyl donor, plays a central role in cellular processes such as DNA, RNA, and protein methylation, as well as metabolic pathways like polyamine synthesis and the transsulfuration cycle. These diverse functions establish its importance in cancer development and progression. Since SAMe discovery more than 70 years ago, extensive research has clarified SAMe’s biological roles, particularly its involvement in methylation and metabolism and its dysregulation in cancer. SAMe depletion has been identified as a hallmark of many cancers, and enzymes involved in its metabolism have been identified as tumor biomarkers [9,61]. Altered MAT1A, MAT2A and MAT2B expression, as well as SAMe/SAH ratio, potentially provide diagnostic and prognostic insights.

SAMe’s relationship with cancer has been explored in a plethora of cancers, both investigating its implication in cancer establishment and progression and as a potential therapeutic agent. Despite the wide variety of malignancies studied, many of the works just employ tumoral cell lines but lack validation of the results in preclinical animal models. Moreover, several of the animal experiments have been performed using xenograft models by injecting the previously in vitro tested tumor cell lines into immunocompromised mice [254,259,261]. Although these kinds of animal models are very useful, the validation of discoveries and treatments in preclinical animal models developing the specific type of cancer studied may more reliably resemble the pathology that occurs in humans and would be more closed to the clinical findings.

Regarding therapeutic strategies involving SAMe supplementation, overexpression or inhibition of methionine cycle enzymes (MAT1A, MAT2A) [177,250,259] and synergistic therapies with chemotherapeutic agents have shown significant promise in preclinical studies. Moreover, SAMe-based treatments have demonstrated potential in restoring methylation homeostasis, overcoming chemoresistance, and enhancing apoptosis in various cancers, including HCC, CRC and breast cancer. However, translating these preclinical successes into effective clinical applications remains challenging due to variability in SAMe’s effects, delivery issues and a lack of tailored patient stratification. This absence of precise biomarkers to obtain patient subtyping avoids the effective transfer of treatments from the bench to the bedside, as demonstrated by the few clinical trials conducted and the low success achieved [249]. In addition, there are other points that should be addressed for the obtention of an effective SAMe treatment, including the inefficient delivery of SAMe due to low bioavailability and the paradoxical outcomes that can be obtained (e.g., excessive methylation) derived from context-dependent effects.

Always keeping in mind those limitations, the most promising emerging area would be the targeting of methionine cycle enzymes to regulate SAMe availability in cancers that exhibit methionine dependency, such as CRC and lung cancer [177,259]. Similarly, exploring epitranscriptomic regulation (e.g., m6A modifications) mediated by SAMe and its interaction with non-coding RNAs opens new opportunities for understanding tumor progression and resistance mechanisms [157,241]. Precision medicine approaches focusing on individual tumor profiles and integrating SAMe-targeted strategies with chemotherapeutics would increase the effectiveness of cancerous treatments. In our opinion, combination therapies taking advantage of SAMe’s ability to enhance chemosensitivity and modulate epigenetic status will be the most promising, as SAMe combined with decitabine or doxorubicin in targeting breast cancer, with cisplatin in head and neck squamous cancer, or even sensitizing cancer cells to radiotherapy [146,254,255,257,258].

In summary, the modulation of SAMe metabolism broadly found in many kinds of different cancers makes it a potential target for therapy, mainly in combination with other chemotherapeutics as a sensitizing agent. Nevertheless, important concerns regarding among others precision or personalized medicine approaches must be addressed before using it in clinical practice, improving therapeutic outcomes across a broad range of malignancies.

## Abbreviations

5-MTHF	5-methyltetrahydrofolate
5,10-CH_2_THF	5,10-methylene-THF
TOP	5′-terminal oligopyrimidine
ACSL3	acyl-CoA synthetase long chain family member 3
AFP	alpha-fetoprotein
AK4	adenylate kinase 4
AKT	protein kinase B
ALT	alanine transaminase
AMD	SAMe decarboxylase proenzyme
AMD1	SAMe decarboxylase proenzyme 1
AMPK	AMP-activated protein kinase
APEX1	apurinic/apyrimidinic endonuclease 1
AST	aspartate transaminase
ATF3	activating transcription factor-3
ATP	adenosine triphosphate
BHMT	betaine Hcy methyltransferase
CBS	cystathionine beta-synthase
CDAA	choline-deficient L-amino acid-defined
CDK2	cyclin-dependent kinase 2
cFLIP	cellular FLICE inhibitory protein
CIMP	CpG island methylator phenotype
CRC	colorectal cancer
CTH	cystathionase
dcSAMe	decarboxylated SAMe
DILI	drug-induced liver injury
DNMTs	DNA methyltransferases
DNMT1	DNA methyltransferase 1
DOK7	downstream of kinase 7
DUSP1	dual-specificity MAPK phosphatase
EGF	epidermal growth factor
eIF5A	eukaryotic initiation factor 5 A isoform 1
ELOVL2	elongation of very long chain fatty acids-like
eNOS	endothelial nitric oxide synthase
ERα	estrogen receptor α
ERK	extracellular signal regulated kinase
ERRFI1	ERBB receptor feedback inhibitor 1
ESR1	estrogen receptor 1
GIT1	G Protein Coupled Receptor Kinase Interacting ArfGAP 1
GNMT	glycine N-methyltrasferase
GSH	glutathione
*GSTπ*	glutathione-S-transferase
H3K4	histone H3 fourth lysine
H3K27	histone H3 27th lysine
H-Ras	HRas proto-oncogene
HCC	hepatocellular carcinoma
HFD	high fat diet
HGF	hepatocyte growth factor
HuR	Hu antigen R
ID4	inhibitor of differentiation 4
IGF	insulin-like growth factor
IL-1β	interleukin-1β
IHS	isolated hepatic steatosis
JAK	Janus kinase
KTMs	lysine-specific methyltransferases
LARP1	La-Related Protein 1
LDH	lactate dehydrogenase
LINE-1	long interspersed nuclear element 1
LKB1	serine/threonine protein kinase 11
m^6^A	N^6^-methyladenosine
MAPK	mitogen-activated protein kinase
MASH	metabolic disfunction-associated steatohepatitis
MASLD	metabolic disfunction-associated steatotic liver disease
MAT	methionine adenosyltransferase
MAT I/III	methionine Adenosyltransferase I/III
MAT1A	methionine adenosyltransferase 1A
MAT2A	methionine adenosyltransferase 2A
MAT2B	methionine adenosyltransferase 2 non-catalytic beta subunit
MCD	methionine and choline deficient
MDR1	multidrug resistance 1
MeCP2	methyl-CpG-binding protein 2
MEK	mitogen-activated protein kinase kinase
METTL3	methyltransferase-like protein 3
METTL14	methyltransferase-like protein 14
MGMT	O(6)-methylguanine DNA methyltransferase
MMP-2	matrix metalloproteinase-2
MS	methionine synthase
mSAMC	mitochondrial S-adenosylmethionine carrier
MTs	methyltransferases
MTA	methylthioadenosine
MTAP	MTA phosphorylase
MTHFR	methylenetetrahydrofolate reductase
mTOR	Mammalian target of rapamycin
MLH1	MutL Homolog 1
NAFLD	non-alcoholic fatty liver disease
NAM	nicotinamide
NAT1	N-acetyltransferase type 1
NNMT	nicotinamide methyltransferase
ODC	ornithine decarboxylase
PC	phosphatidylcholine
PE	phosphatidylethanolamine
PEMT	phosphatidylethanolamine N-methyltransferase
PI3K	Phosphoinositide 3-kinase
PITX2	paired-like homeodomain transcription factor 2
PRA	progesterone receptor α
PRMTs	protein arginine methyl transferases
PTEN	Phosphatase and tensin homolog
RASSF	Ras-association domain family/tumor suppressor
RIP1	receptor-interacting Protein 1
ROS	reactive oxygen species
SAH	S-adenosylhomocysteine
SAHH	S-adenosylhomocysteine hydrolase
SAMe	S-adenosylmethionine
SLD	steatotic liver disease
SOCS	suppressor of cytokine signaling
SPD	spermidine
SPM	spermine
STAT	signal transducer and activator of transcription
TCF4	transcription factor 4
TET	translocation methylcytosine dioxygenase
TG	triglyceride
TGF-β	transforming growth factor-β
THF	tetrahydrofolate
TNF	tumor necrosis factor
TROP2	trophoblast surface antigen 2
UCHL1	ubiquitin C-terminal hydrolase L1
uPA	urokinase-type plasminogen activator
VLDL	very low density lipoproteins
WT1	Wilms’ tumor 1
ZEB1	zinc finger E-box-binding homeobox 1
